# Internet Narratives Focused on Health Travelers’ Experiences in India: Qualitative Analysis

**DOI:** 10.2196/15665

**Published:** 2020-05-14

**Authors:** Joseph Brown, James Johnson, Margaret E Ozan-Rafferty, Manoj Sharma, Salvatore Barbera

**Affiliations:** 1 The Herbert H & Grace A Dow College of Health Professions Central Michigan University Mount Pleasant, MI United States; 2 Blessing Health System Quincy, IL United States; 3 Covenant HealthCare Saginaw, MI United States; 4 Health Administration Management Program College of Business Florida Atlantic University Boca Raton, FL United States

**Keywords:** medical tourism, India, global health, personal narratives, travel-related illness, qualitative research, patient satisfaction, delivery of health care, travel, data collection

## Abstract

**Background:**

The medical tourism industry is currently popular in India, but there is no confirmation of the common perspectives among the country’s medical travelers.

**Objective:**

This qualitative research study analyzed web-based narratives from health travelers visiting India and described the themes of their experiences. This study aimed to answer the following primary question: What can we learn about health travelers’ experiences in India from an analysis of their web-based narratives? The secondary questions were as follows: (1) What are the primary health care reasons for which patients in the examined narratives traveled to India? (2) What can be derived from the narratives regarding medical tourists’ satisfaction with the outcome and result of the treatment they received in India? (3) What are some positive and negative factors influencing medical tourists’ perceptions and overall experiences about their health travel to India? (4) What are the characteristics of medical tourists who write web-based narratives regarding their health experiences in India?

**Methods:**

Publicly available narratives written by medical tourists who visited India were obtained from a Google search. The narratives included blog posts and discussion board posts by medical tourists. The analysis process consisted of initial open coding being conducted on the narratives to create initial codes and identify common themes with a focus on the primary research question and subquestions.

**Results:**

Although Mumbai, Chennai, and New Delhi were not the only destination cities mentioned, these were the most popular cities patients visited for care. The medical tourists, who stated their origin country, came from one of the following continents: Africa, Europe, North America, and Oceania. Dental care, Ayurveda treatment, and eye care were the most popular types of care that medical tourists sought. The results showed that most of the medical tourists were happy with the overall experience of receiving care in India. The most popular themes with regard to the patients’ satisfaction were low costs, good customer service, and services being offered that were unavailable in their home country. When negative feedback was provided, it was mainly concentrated on the overall environment of India being unorganized and unsanitary.

**Conclusions:**

Primarily, the study’s findings can benefit health care providers and patients. Providers hosting medical tourists in India can use negative feedback to improve their services; similarly, providers who are losing patients to medical tourism can identify opportunities for improvement (ie, why are we losing patients). Indian providers hosting medical tourists should keep their prices competitive and continue to provide exceptional service; however, they should do their best to lessen the crowdedness of their facilities while making sure they are esthetically pleasing. Providers losing patients to medical tourism need to identify ways to ensure their services match the benefits that their international counterparts are providing, such as competitive pricing and expansion on the services provided.

## Introduction

### Background

Depending on the source, the terms “health tourism,” “medical tourism,” and “wellness tourism” are used very loosely and unsystematically [1]. According to Connell [2], this is most likely because the boundaries between these terms are unclear. For the purposes of this paper, the terms medical tourism and health traveler are used to define instances when a patient travels across international borders to obtain medical care. These medical tourism treatments may range from highly invasive heart surgeries to less invasive procedures such as dental work. Mutalib et al [3] stated that “medical tourism (specifically) is not a new phenomenon, what is new is actually the trend of practicing medical tourism.” Lunt et al [4] explained that availability, cost, expertise, and cultural and familial reasons motivate patients to seek care abroad. The majority of medical education in India is subsidized by the government; therefore, the doctors do not have the heavy burden of student loans [5]. This helps to drive down the cost of care. Similarly, Alsharif et al [6] examined medical tourists visiting India, China, Jordan, and the United Arab Emirates and found that the most important reasons for patient travel were cost, provider’s reputation, and hospital accreditation. Throughout the paper, the terms posts, narratives, and journals have been used interchangeably to represent the qualitative data analyzed to execute this research.

The internet can play a role in a prospective patient’s medical tourism decisions. According to Lunt et al [4], patients consult a range of information sources before making decisions, and the internet plays a key role in providing information from informal networks. Consumers participating in web-based health care communities often reveal personal information in intricate detail [7]. As it relates to health care specifically, trust in the destination assures medical tourists who choose to visit a particular destination that the services provided will be transparent, reliable, and risk and hassle free [8]. According to Lunt et al [4], medical tourists often pay more attention to soft information than hard clinical information.

### Prior Work

This study originated from the recommendations in the further research section in the study by Ozan-Rafferty et al [9] titled “A qualitative analysis of internet narratives by health travelers to Turkey”. Her study analyzed web-based narratives from health travelers to Turkey and described the themes of their experiences. This study will mimic the established method of the abovementioned study but focus on India instead of Turkey.

### Setting

India is roughly one-third the size of the United States but its population is about four times that of the United States [10]. India’s private sector providers, along with independent medical tourist–facilitating organizations, have taken the lead in developing medical tourism [11]. The combination of India’s economic liberalization, growing middle class, and the rise in medical tourism has contributed to the growth in the number of private, for-profit hospitals [12]. The primary reason for India’s growing medical tourism sector is cost savings. On average, medical services provided in India cost almost half the price of those offered by any other developed country [13]. The Indian government has encouraged the growth of medical tourism by issuing special visas for medical tourists [6]. In addition, many patients are intrigued by the possibility of combining their medical treatment with a vacation [2].

There are no authoritative data on the number and flow of medical tourists between nations and continents [14]. This is mainly because there are no international standards for the transmission of information to clinicians in the patient’s country of origin [15]. Bernasek [16] cited the World Health Organization’s projection that 16 million Americans would travel abroad for medical care in 2017. In their study on medical tourism growing in the Indian health care market, Gupta et al [17] noted that India’s Ministry of Tourism has even conducted road shows in West Asia (Dubai, Riyadh, Kuwait, and Doha) to promote medical tourism.

Similar to the research by Ozan-Rafferty et al [9], this research assumes that understanding the experiences of medical tourists will benefit the providers who service current and future medical tourists. In addition, the findings from this study can be used to assist in decision making for patients considering health travel in the future, and they may strengthen health administration education by providing insights on medical tourism [9]. In addition, this study’s outcomes may lead to the development of more online communities focused on improving medical tourism. In the future, this study can help further the push of establishing a global tracking system for statistics on the number of medical tourists. More importantly, we hope that this study will encourage more research on the topic of medical tourism by way of its recommendations and drive competition resulting in a better health experience for all patients irrespective of their location.

No funding was required for this study. As per Central Michigan University’s Institutional Review Board (2017), it has been determined that this project does not meet the definition of human subject research under the purview of the institutional review board (ie, exempt status). This is because no human subjects were involved in the research, and no identifiable information was included.

### Research Questions

The medical tourism industry is currently popular in India, but there is no confirmation of the common perspectives among the country’s medical travelers. The questions to be answered are as follows: (1) What are the primary health care reasons for which patients in the examined narratives traveled to India? (2) What can be derived from the narratives regarding medical tourists’ satisfaction with the outcome and the result of the treatment that they received in India? (3) What are some positive and negative factors influencing medical tourists’ perceptions and overall experiences about their health travel to India? (4) What are the characteristics of medical tourists who write web-based narratives regarding their health experiences in India?

## Methods

### Recruitment

This is a qualitative research study based on a narrative analysis from Creswell’s qualitative approach [18]. Narratives written by medical tourists visiting India were obtained using publicly available webpages identified via a Google search. The narratives included blog posts and discussion board posts by medical tourists who visited India for care. Data were extracted using purposeful sampling, which focuses on information-rich cases related to a specific topic [19]. Similarly, Blumberg et al [20] stated that purposeful sampling is when researchers select participants arbitrarily for their unique characteristics or their experiences. In this case, the focus was on patients who left their home countries and received care in India. The search terms used included, but were not limited to, “health travel,” “medical tourism,” “plastic surgery,” and “wellness travel” and incorporated “India,” “Mumbai,” “New Delhi,” and “Kolkata.” Specific clinical procedures used in the search were determined based on the reviewing sites that promoted health travel to India, as well as findings from the literature review, and terms noted in initial findings.

Results from the first 10 pages of the general Google search were reviewed to find narratives that met this study’s criteria, which were as follows: (1) a first-person narrative written by an individual who underwent treatment or their partner on the trip, (2) a narrative written in English, (3) a narrative that includes a description of the type of procedure, (4) a narrative that includes a personal experience of health travel to India, and (5) a publicly available narrative that does not require a password or discussion board membership to be accessed [9]. Then, following the core of the process established in the “A qualitative analysis of internet narratives by health travelers to Turkey” study, the next step limited the search results to only include discussions. Next, results from the first 10 pages of the “discussion” were only filtered results that were reviewed to determine whether there were any eligible narratives meeting the same 5 requirements listed earlier.

On the basis of the research by Ozan-Rafferty et al [9], the following review attributes were considered when collecting the narratives. Any narrative that was longer than a single sentence was added to the queue for analysis. However, some of the narratives were in a chronological story format, and some were shorter in length and outlined a specific part of a general larger experience [21]. Even though posts and blogs related to the topic of the author’s health travel were included in the research, other topics written by the author were reviewed to obtain demographic or characteristic information such as the author’s age, gender, or country of origin. Multimedia Appendix 1 displays the steps in the narrative process by way of a flow diagram.

On the basis of the types of medical tourism treatments identified in the research review, 256 different keyword searches were performed. These produced more than 800 million search results. Of course, this is when counting results falls outside of the first 10 pages. After the filtering process was completed, there were 53 narratives available for analysis.

### Coding Analysis

For the coding process, the identified narratives were copied from the internet and posted in individual Microsoft Word (Microsoft Corporation) documents. Each document was named by using an identifier (ie, “000”) that coincides with its entry number in the process and findings log. Similar to the study by Ozan-Rafferty et al [9], the narratives that included more than one entry were collected chronologically from the oldest to the newest. All the narratives combined totaled to 121 pages. The largest page count of a single narrative used was 23. There were 34 narratives that were 1 page each. As each narrative was contained in its own document, the page count was rounded off. For example, a narrative that was one and half pages long was counted as being 2 pages long.

Initial open coding was conducted on the narratives to create initial codes and identify common themes with a focus on the primary research question and subquestions [9]. The unit of reference was a sentence [21,22], and a total of 2536 units of measure were analyzed. In addition, the content of each sentence was examined to identify positive and negative opinions [22]. Written words were analyzed using narrative analysis theory principles to create primary (parent) and secondary (child) themes [9]. As the analysis occurred, notes were taken to clarify the concept behind some of the codes and sentences being analyzed. More than 40 primary themes were identified, and if necessary, memos were kept for each sentence coded to add context and keep track of concepts.

A second review was completed, and the narratives were entered into NVivo Pro Version 11 (QSR International) where the initial codes, key concepts, and themes identified in the open coding were clustered [23]. As was performed in the study by Ozan-Rafferty et al [9], NVivo was used to identify relationships with the various initial themes. Each sentence along with its corresponding code was entered into NVivo. The codes were clustered into categories, and various clusters were used to identify trends and relationships.

A final round of selective coding [23] was completed by scanning all the data and codes and reviewing all themes. Multimedia Appendix 2 displays the theme frequency count table. To confirm coding accuracy, an additional researcher was approved by the dissertation committee, and he randomly selected 8 of the 53 narratives to review. For the random selection process, the narratives were numbered 1 to 53 but were in no specific order. The 8 narratives selected were independently coded by the additional researcher. After comparing the additional researcher’s codes with the primary researcher’s initial codes, there was only 96.5% reliability (reliability=number of agreements divided by the number of agreements + disagreements). It is important to note that any personal identification details of the narrator were removed and not considered in this research.

As has been mentioned, there was a committee outside of the additional researcher. This committee consisted of 3 members, and they approved all major components of the study. Given that she had prepared a similar project focusing on Turkey, Dr Ozan-Rafferty was the committee member who assured that the methods were followed correctly. Dr Sharma, originally from India, assured the accuracy of information depicted in the narratives, and Dr Johnson was the chairperson overseeing all aspects of the project.

### Data Exclusion

During the search process, it was not very difficult to find weblinks in which patients were discussing their medical experiences in India. However, most of the initial findings were not eligible for this study because they were being posted for promotional purposes. For example, the direct quotes were on the webpages of hospitals that wanted people to come to their facilities for a certain procedure. The articles that quoted medical tourists but were not written by the actual medical tourists or by someone who went on the trip with them were excluded. There were many photo blogs and video blogs (vlogs); by strictly following the method established in previous research on this topic, this type of media was not considered. Adding a photo, video, or the aspect of judging a person’s tone could have easily distracted from the overall word analyzation, which was the primary focus of this project. It was not uncommon to find blogs or articles written by doctors who treat medical tourists. If joining a forum or discussion group was required to obtain any narratives, obtaining membership in the discussion forum or discussion group was not done to ensure all information used is truly publicly available. A log of each narrative was created and included the web address of each post. Some medical tourists posted the exact copy of the narrative on multiple webpages, and those duplicates were eliminated from this research.

## Results

### Overarching Narrative Details

The entire search process took place from November 5 to 16, 2017. Narratives were reread on the web on January 8, 2018, to confirm no additional postings were added. Some search inputs garnered millions of results. Of the narratives that were used, most came from discussion forums where someone would start a thread such as, “Has anyone been to India for…,” and previous medical tourists responded by sharing their stories and helping out other prospective health travelers by answering general questions. There were several blogs as well. Some of the blogs were very professional-looking (with photos and embedded videos), whereas others were simple text entries. From time to time, a narrative was found in the comments of an article about medical tourism, so it was important to read all the comments on an identified weblink. At the conclusion of the search, a total of 53 narratives were deemed eligible for analysis. Of those 53 narratives, 6 were blog posts. The remaining 47 narratives were from a discussion thread.

Of the 53 narratives collected, there were only 8 narratives in which the medical tourists’ countries of origin (home countries) were unidentifiable. Table 1 displays the continent of origin and country of origin for each patient. This table is in an alphabetical descending order. With 11 narratives, the United States was the country with the highest number of medical tourists visiting India, followed by the United Kingdom and Australia (10 each). There were 3 countries with only 1 medical tourist visiting India: Morocco, Austria, and France.

**Table 1:** Health travelers’ country of origin.

Country		Travelers, n
**Africa**		**1**
	Morocco	1
**Europe**		**16**
	Austria	1
	France	1
	Germany	2
	Ireland	2
	United Kingdom	10
**North America**		**18**
	Canada	7
	United States	11
**Oceania**		**10**
	Australia	10
**Undetermined**		**8**
	Undetermined	8
Total		53

### Narrative Content Details

Of the narratives analyzed, the most frequent procedure availed by a medical tourist was some kind of dental care. This was followed by Ayurveda treatment and Lasik eye surgery. There were a number of services that were only mentioned once across all the narratives. These services ranged from gallbladder surgery to a hair transplant. Although it did occur, it was not a common trend for patients to avail more than one medical service while in India. Table 2 consists of the detailed counts of how often a procedure was availed.

Tables 3-6 provide details regarding each patient and demographic details about where the procedures occurred. With regard to gender, 21 of the medical tourists were men and 18 were women; the gender of the remaining 14 tourists was unidentifiable. In most cases (32 narratives), there was no indicator of the patient’s age. Regarding the identified ages, it is notable that 14 of the patients were older than 40 years when their initial post was made. Then, 16 of the narratives referred to a person accompanying the medical tourist while in India, and of those 16, 4 were authored by the person who accompanied the patient. Only 4 narratives mentioned that the patients used a medical tourism–facilitating organization.

Most of the narrators (n=30) only made a single post, and 11 made two posts. One narrator made 21 entries, and another made 39 entries. There were 4 narratives in which years passed between the first and last posts. However, all the other narratives consisted of posts that occurred within a year or another. In addition, 45 of the posts were made between 2010 and 2017. The oldest post analyzed was from March 2005, and the newest post was from December 2017. A total of 29 narratives mentioned the actual treatment date.

The destination city was noted in 38 of the narratives. Mumbai, Chennai, and New Delhi were the most popular cities that patients visited for care. Regarding specific facilities, 23 different facilities were listed, but only 2 were listed more than once: Asian Joint Reconstruction Institute of Chennai and MY EYE.

The authors were very open about their experiences. They shared details such as the food they ate all the way up to their personal fears (such as flying). It was similar to reading a personal journal. From a personality standpoint, very few users described themselves, but when they did, they included descriptors such as humble, self-conscious, and focused on health. Most narrators had a positive attitude rather than a negative one. This is because they stated things such as “I’m excited about the trip” or “I had been looking forward to this.” Many sentences that were coded described the narrator as not having a complaining attitude, having an overall positive view on life, or being optimistic about their health’s improvement after receiving treatment in India. Contributors also encouraged others. For example, a patient with Crohn disease sent this message to another person diagnosed with Crohn disease: “I really hope she feels better, and I know sometimes it can feel like there is no hope and you're willing to try everything to help alleviate the symptoms.” The following sections are reasons for medical tourism, patient satisfaction, and perception influencers. These are used to highlight the significant themes identified in the narratives.

**Table 2:** Health travelers’ primary and additional procedures.

Row labels	Count of primary procedure^a^	Count of additional procedures
Ayurveda	9	N/A^b^
Dental	13	N/A
Eyelid surgery	1	2
Gallbladder surgery	1	3
Gynecomastia surgery	1	N/A
Hair transplant	1	N/A
Heart surgery	1	N/A
Hernia surgery	1	N/A
Joint replacement	3	N/A
Lasik eye surgery	8	2
Limb lengthening	3	N/A
Physical exam	2	N/A
Physiotherapy	1	N/A
Plastic surgery	3	1
Sex reassignment surgery	1	N/A
Shot	1	N/A
Spine surgery	1	N/A
Surgery	1	N/A
Surrogacy	1	N/A
Grand total	53	8

^a^Primary procedure is the procedure the narrator noted first or mentioned as their primary reason for traveling to India for care.

^b^N/A: not applicable.

**Table 3:** Health travelers’ date of initial narrative, demographics, location of treatment, date of treatment, facilitator, facility, and accompanying person.

Author	First post	Gender	Age of patient when post was made (years)	City of procedure	Treatment date	Facilitator	Facility	Accompanying person
001	August 15, 2016	Female	N/A^a^	N/A	N/A	N/A	N/A	N/A
002	November 11, 2010	Male	N/A	New Delhi	2010	N/A	N/A	N/A
003	March 29, 2017	Male	60	Chennai	January 16	N/A	Asian Joint Rebuilding Institute in Chennai	N/A
004	N/A	Female	N/A	Goa	N/A	N/A	Vrundavan Hospital	Spouse
005	July 28, 2010	Male	50-64	Delhi	February 12	SCODE	N/A	N/A
006	July 10, 2016	Female	N/A	New Delhi	N/A	N/A	Delhi Dental Center	N/A
007	January 17, 2010	Female	50-64	Margao	2009	Apollo Victor	Apollo Victor	Daughter
008	April 2, 2016	N/A	N/A	Hyderabad	N/A	N/A	Dr. Motiwala Dental Clinic	N/A
009	June 11, 2016	Female	N/A	Hyderabad	N/A	N/A	N/A	N/A
010	May 20, 2015	Female	50-64	Hyderabad	2014	N/A	Dr. Motiwala Dental Clinic & Implant Center	N/A
011	December 4, 2017	N/A	N/A	Chennai	2017	N/A	Rajan Dental	Husband
012	April 15, 2013	Female	50-64	Nuvem	December 11	N/A	MY EYE	N/A
013	November 1, 2013	Female	35-49	Mapusa	N/A	N/A	N/A	N/A
014	November 5, 2013	Male	50-64	Goa	November 4, 2013	N/A	N/A	N/A
015	December 10, 2014	N/A	N/A	N/A	2015	N/A	N/A	N/A
016	January 1, 2008	N/A	N/A	Chennai	February 27, 2008	N/A	Sankara Nethralaya Hospital	N/A

^a^N/A: not applicable.

**Table 4:** Health travelers’ date of initial narrative, demographics, location of treatment, date of treatment, facilitator, facility, and accompanying person.

Author	First post	Gender	Age of patient when post was made (years)	City of procedure	Treatment date	Facilitator	Facility	Accompanying person
017	November 3, 2016	Male	58	Chennai	October 15	N/A^a^	Asian Joint Rebuilding Institute in Chennai	Wife
018	July 28, 2010	Female	N/A	N/A	N/A	N/A	N/A	N/A
019	September 13, 2013	N/A	N/A	N/A	N/A	N/A	N/A	N/A
020	January 7, 2014	N/A	N/A	N/A	N/A	N/A	N/A	N/A
021	August 4, 2009	Female	35-49	Margao	November 8	N/A	MY EYE	N/A
022	June 19, 2014	N/A	N/A	N/A	June 13	N/A	N/A	N/A
023	June 25, 2010	N/A	N/A	N/A	N/A	N/A	N/A	Other
024	July 3, 2016	Female	70	Kolkata	2014	N/A	N/A	Family
025	May 3, 2016	Male	N/A	Chennai	May 14	N/A	Asian Joint Rebuilding Institute in Chennai	Wife
026	September 27, 2015	Male	35-49	New Delhi	September 15	N/A	ReLEX Smile Lasik	N/A
027	November 24, 2012	Male	35-49	Mumbai	2012	N/A	Hinduja Hospital	N/A
028	January 23, 2012	Male	20-29	N/A	2010	N/A	N/A	N/A
029	January 14, 2010	Male	N/A	Mumbai	N/A	N/A	Saifee Hospital	N/A
030	August 8, 2015	N/A	N/A	Palakkad	2015	N/A	N/A	N/A
031	September 25, 2007	Male	40	Bangalore	N/A	N/A	N/A	N/A
032	March 24, 2017	Female	N/A	Mumbai	2008	N/A	N/A	Father
033	March 12, 2005	Male	N/A	Thiruvananthapuram, New Delhi	N/A	N/A	N/A	N/A
034	May 26, 2005	Male	35	Panaji	N/A	N/A	N/A	Wife

^a^N/A: not applicable.

**Table 5:** Health travelers’ date of initial narrative, demographics, location of treatment, date of treatment, facilitator, facility, and accompanying person.

Author	First post	Gender	Age of patient when post was made (years)	City of procedure	Treatment date	Facilitator	Facility	Accompanying person
035	September 30, 2009	N/A^a^	N/A	N/A	N/A	N/A	N/A	Family
036	February 23, 2010	Male	47	New Delhi	April 16, 2010—gallbladder surgery; April 13, 2010—dental; April 14, 2010—physical exam; April 23, 2010—dental; April 2010— colonoscopy	N/A	Apollo Hospital Escorts Heart Institute	N/A
037	August 12, 2015	Female	49	N/A	N/A	N/A	N/A	N/A
038	April 20, 2016	Male	N/A	N/A	April 16	IndianMedTrip Health Care Consultants	N/A	Sister
039	July 5, 2014	N/A	N/A	Mumbai	June 25, 2014	N/A	Mangal Anand Center	N/A
040	September 4, 2013	Female	50-64	Goa	February 1	N/A	N/A	Husband
041	February 24, 2016	Male	N/A	Pune	February 2, 2016	N/A	N/A	N/A
042	December 25, 2013	Male	27	N/A	2013	N/A	N/A	N/A

^a^N/A: not applicable.

**Table 6:** Health travelers’ date of initial narrative, demographics, location of treatment, date of treatment, facilitator, facility, and accompanying person.

Author	First post	Gender	Age of patient when post was made (years)	City of procedure	Treatment date	Facilitator	Facility	Accompanying person
043	July 21, 2015	N/A^a^	N/A	N/A	N/A	Placidway	N/A	Spouse
044	April 17, 2014	Female	N/A	Bangalore	N/A	N/A	N/A	Daughter
045	November 21, 2013	Female	N/A	Mumbai	N/A	N/A	Rotunda—The Center for Human Reproduction	Husband
046	December 9, 2015	Female	N/A	Kartikulam	December 15	N/A	Ayurveda Yoga Villa	N/A
047	July 29, 2015	Female	41	Coimbatore	July 15	N/A	Vaidyagrama	N/A
048	March 19, 2012	Female	N/A	Coonoor, India	January 12	N/A	Ayurveda Yoga Retreat	N/A
049	September 12, 2012	N/A	N/A	N/A	N/A	N/A	N/A	N/A
050	December 4, 2006	Female	45	N/A	N/A	N/A	N/A	N/A
051	March 25, 2010	Female	N/A	Puttaparthi	N/A	N/A	N/A	Other
052	September 26, 2010	Male	N/A	Una	N/A	N/A	N/A	N/A
053	July 1, 2015	N/A	N/A	Mysore	N/A	N/A	Columbia Asia Hospital	N/A

^a^N/A: not applicable.

### Reasons for Medical Tourism in India

The medical tourism drivers were patients attempting to receive the best care for a specific diagnosis, travelers trying to overcome home country barriers (ie, costs or unavailability), and patients seeking alternative treatments for a condition; there were a few who were just curious about the overall medical tourism experience and options. One of the more polarizing direct quotes was “No, I can't get the same treatment in America, that's why I'm here.” Another stated “I have private health insurance, but I wasn’t happy with the experience (or lack of) surgeons have here in carrying out the procedure.”

It appeared that patients became open to the idea of medical tourism while researching one of two things. They were either looking for a provider who specialized in treating a certain diagnosis or researching procedures to treat certain conditions. A patient exploring Lasik options stated:

At 64 I decided that I had enough of broken glasses reading in bed. I went for a consult with a very reputable eye laser company here in Toronto. After all the tests, (no blood work) they can only restore my far vision at the cost of $6000+. I did some research and found a doctor in India…made an appointment through their site.

In fact, there have been studies that confirm various health clinics will use the internet to advertise their services globally and that they not always clearly depict the risks of certain treatments [24].

#### Patient Satisfaction

The number of satisfied health travelers greatly outnumbers those who had complaints or expressed unhappiness. For example, some narrators made comments such as “happy with dental implants,” “normal life regained,” or just “happy with results.” General problem resolution comments were the most abundant indicator that patients were satisfied with the care they received. With Ayurveda treatments, specifically, there were comments such as “found a love for yoga” or “found true self” that were not initially expected outcomes by the narrator. One Ayurveda patient said they found an “unexpected healing.” A narrative, in which a patient received Lasik eye surgery, stated:

My sight is amazing and having had a session with Dr. Chandrakant Gaonker again this morning, he says it will get better each day.

Another critical point surrounds the topic of desired repeat travel to receive care again. The repeat travel was not because the initial visit required any type of follow-up care, but it was because the patient was so happy with the experience, they wanted to go back to get another procedure. There was a narrator who mentioned they would only be getting their dental work completed in India moving forward. In addition, there was a narrator who considered permanently moving to India because they felt the care was highly superior to that offered in their home country for supporting specific chronic conditions.

When patients expressed negative feedback on the outcome and results, the negativity fit in the following primary categories: general bad experience, bad appearance, unresolved problem, or issue worsened. Dental procedures most frequently garnered an unsatisfactory comment about the results, especially when relating to a narrator’s appearance after care was given. After receiving a dental bridge, a patient said:

The curve of my finished bridge was way out of sync with my upper gum line, and the inner gum line sat too low from my gum, causing every bite I took to clog up in that space.

One patient with Crohn disease did not mention that their condition worsened, or any corrections had to be made, but that the Ayurveda treatment had no impact:

Despite the doctor’s assurances, it did not help much, I still had the same intolerances and same degree of inflammation.

#### Perception Influencers

The influences that shaped the narrators’ Indian medical tourism care experience included India’s general environment. This was the noise, smells, and basic laws. One patient summarized his feelings by saying:

The problem is that I don’t know the culture, don’t speak the local dialect, have a hard time understanding the English of the average Indian, am a little intimidated by the system, and feel somewhat alone and isolated.

Cost was another perception influencer; the costs mentioned were everything from the lodging to receiving care. Travel logistics (such as the passport and visa process), quality of care, customer service, facility condition, and follow-up care were the other main contributors that shaped narrators’ opinions. For example, regarding costs, a notable trend among the narratives was that they described the payment process. Prepayment was one of the more frequent payment processes mentioned. Throughout all the narratives, there were 6 references to a prepayment requirement (before services were rendered). There was one narrator who mentioned they not only had to pay in advance, but they were required to pay cash:

He insists on only cash, so I handed over Rs. 98,500 (remember Rs. 1500 was paid already for the tests). I felt like I was doing some sort of underhand deal, however I fully trusted Dr. Sandip.

## Discussion

### Principal Findings

Almost all the narratives came from patients seeking resolution of a health issue. There were no patients who went to India merely for exploratory purposes. Patients either felt they needed to fix their appearance (ie, cosmetic) or had a serious medical diagnosis such as a hernia. The patients found India by looking for not only the best care but also the most affordable resolution, which is consistent with the findings of the study by Ozan-Rafferty et al [9].

The general theme of all the narratives reviewed was positive. It was rare for a patient to indicate that their travel to India for care was negative. Many of the narratives focused on how affordable the care was when compared with that in the patients’ home countries. An author even mentioned that he tried to give a financial tip to a provider because the care was so good and affordable. The other positive influencers were the narrators’ good interactions with the health care providers in India, the modern facilities, and the resolution of their health issues. Many narrators mentioned that they would be a medical tourist in India again and encouraged others to travel to India for medical care. With that said, it is still important to state that there could be far more negative experiences, but the patients did not necessarily document their experiences on the web.

#### Reasons

The narrators said that a barrier prevented them from resolving their health issues in their home country and drove them to India. Some people sought procedures such as Ayurveda treatment, Desarda hernia repair, or limb lengthening that are simply not offered in all parts of the world. Some patients could not afford or did not have the type of insurance necessary to get the care they needed. A good example is the patients who went to India for dental care because they did not have dental insurance in their home countries. In fact, dental was the most common type of care the patients in this study received. There was an author who said they received a root canal at no charge.

The blog comments and discussion threads included detailed conversations between former and possible future patients just talking about medical tourism in general. It was obvious that some participants provided more insights to the readers in a more private format because they would reference a private message exchange with another user. Sometimes experiences were reconfirmed when other medical tourists commented that they had gone to India for care and had a similar story (ie, same doctor or facility).

#### Satisfaction Levels

As mentioned, many of the authors said their primary issue was resolved for an affordable price, and the authors encouraged others to consider medical tourism when looking to have their health issues resolved. As this study is replicated, the number of primarily negative-toned narratives is critical, similar to those highlighted in the next section; it will help to answer the question, if providers of medical tourism are improving (based on feedback) or ignoring opportunities to get better. In addition, patients’ opinions, which were once positive, could change if there are any delayed side effects.

#### Negative Viewpoints

All of the negative comments tied back to the health issue not being fixed to the patient’s satisfaction. This section will focus on two of the narrators who were the most dissatisfied. A dental patient’s condition worsened as a result of bad implants, and the author realized it would be difficult for her to file a lawsuit because she voluntarily went out of her home country to get care. Another narrator said that providers spoke to each other in an unidentified language even though they all spoke English, and this made her feel excluded and uncomfortable. That same patient said she believed some of the utensils used during preoperational procedures were not clean.

#### Interactions With Hospitals and Providers

The number of patients and their companions who reported good encounters with the providers in India greatly outnumbered those who experienced bad encounters. The positive comments included, but were not limited to, providers being well dressed, providers having extensive experience, friendly doctors, and some patients being greeted with flowers upon arrival. There were more than a few times that a provider had good web-based reviews, which initially led the patient to the provider. In addition, patients really seemed to be comforted if the provider had experience or training in the United States, and a large number of the patients indicated the providers either spoke English or had a translator readily available.

When negative comments were made specifically related to provider and patient interaction, they seemed to be aimed at inaccurate web-based advertisements of the doctor’s capabilities and specialties and general poor communication. For example, one patient landed in India only to find out their appointment had been canceled, and another narrative described a provider as being money hungry.

#### Challenges to Medical Tourism in India

Some of the main challenges to medical tourism in India are poor visitor experiences expressed in negative web-based reviews, travel logistics (ie, long flights, having to get passports and travel visas), lack of capital compared with other countries marketing medical tourism, and improved health care infrastructure across the globe [13]. Regarding negative web-based reviews, many of the narratives indicated that the patient completed web-based research before deciding to go to India for care, and it was not unusual for patients to remark that it was difficult to identify trustworthy information or find information about medical tourism in India. There were previous medical tourism patients who had posted negative feedback about doctors on web-based forums or blogs and urged others to avoid medical tourism. In the narratives used for this study, a dental patient said the dentist they saw in India had persuasive marketing but was not successful in resolving any of the patient’s primary diagnosis. The patient said the provider deleted any bad reviews users posted on Facebook.

Although India does have a medical tourism–focused visa, the passport and visa process creates another hurdle for patients who want to go to India for care. Once the appropriate passports and visas are obtained, there can be the issue of discomfort owing to a long flight.

India is not the only country known as a medical tourism destination, and some countries are working hard to not lose patients to medical tourism. Within this study, there was a narrative in which a dental patient wrote:

I researched nearly every clinic in New Delhi and trust me—none of them are as well trained and professional as in the U.S. YOU GET WHAT YOU PAY FOR!!! My suggestion is to try Buenos Aires, Argentina.

Brazil, Turkey, Mexico, Costa Rica, and Thailand are just a few of the other countries promoting medical tourism [25]. These options could be more attractive to a potential medical tourism patient if they are closer, cheaper, or do not require a travel visa.

#### Opportunities for Medical Tourism Improvement in India

After reviewing the narratives, three opportunities for the improvement of medical tourism in India are apparent. The first is international accreditation. Several patients did not have a process to ensure they were receiving quality service other than “everything looked up to date and clean.” Although there are hospital-accrediting organizations and processes in India, the certification and approval details need to become more prominent and widely advertised. Adding to that, organizations such as Healthgrades and RateMD could expand to emphasize provider reviews specifically tailored for medical tourists.

There is an opportunity for Indian providers to partner with providers across the globe to assist with patient preparation and follow-up care. Many narratives mentioned that there was additional time spent in India to prepare for their procedure (such as x-rays, bloodwork) or get the appropriate follow-up care. Patients mentioned that they had to do substantial self-care once they went back to their home country, such as changing dressings or preparing food in a certain manner. If an Indian doctor partnered with providers in other countries, this could reassure patients that their needs could easily be met before and after their care was completed in India, eliminating the cumbersome follow-up care process.

The third item is the extremely limited availability of medical tourism insurance that covers the costs of procedures completed overseas. Although the narratives did mention huge cost savings when doing an “apples to apples” comparison (price of procedure in home country vs price in India), the patients were paying out of pocket for travel, lodging, and care. There is a huge opportunity for India to introduce insurance that would help alleviate some of the current medical tourism out-of-pocket costs.

### Limitations

There are no official statistics or databases regarding how many people travel internationally to obtain health care [26]. So, there was no pool of known health travelers that could have been contacted to gather general additional insight or expedite the narrative search process.

Greenhalgh [27] pointed out that a primary limitation of narrative research is that narratives are open to multiple interpretations. With that, Bhattacharya [28] added that researchers need to be mindful of their own subjectivities; they should be aware of how their personal experiences creep into the research process and influence the way in which the researcher understands the narrations.

Following the method established in the study by Ozan-Rafferty et al [9], there were already some built-in limitations. These limitations included the chance for fictitious and biased data to be present in the narratives, and the researcher had limited methods to validate self-reported information [29]. As described in the Methods section, narratives were not used if they were posted for promotional purposes. This would include testimonials posted on the actual webpages of providers, websites that strictly focused on consumers rating their experiences, or any post that flat out said something such as “the provider asked me to post this...” Anonymity releases authors from any consequences of their posts or behavior, but at the same time, anonymity may encourage people to share more on the web and discuss sensitive topics and issues [30]. The sample did not include individuals who lacked access to a computer or some type of internet-connected device (to post their narrative on the World Wide Web) and thus may not be representative of the general population [31]. Of the 53 narratives, only 6 were blogs. As these were not live interactions (ie, real-time interviews or conversations), the researchers were unable to clarify feedback provided by the health travelers [9].

Since Ozan-Rafferty et al [9] completed their original study related to this topic, Google has disabled the search filter that allowed users to limit search results strictly to blogs. The feature was retired in 2014 [32].

### Comparison With Prior Work

It is notable that a portion of this study’s results align with some of the results in the study by Ozan-Rafferty et al [9], which focuses on a completely different country. For example, Europe and North America were the top home origination countries for medical tourists. Most of the patients were content with the care they received while being abroad, and the health travelers thought fondly of the providers they saw in the host countries. One of the negative commonalities that stood out was the bad traffic described in both Turkey and India. The results of studies that follow this same method but apply it to different countries can be combined to make more precise conclusions about medical tourism, overall.

There was a contrast in the popular procedures from each study. In the study by Ozan-Rafferty et al [9], the top procedure that medical tourists availed was hair transplants. However, only 1 patient mentioned getting a hair transplant when India was studied. Ayurveda was the second most mentioned procedure in this study, but it was not mentioned at all in the analysis by Ozan-Rafferty et al [9].

### Conclusions

This is not the first study to focus on medical tourism or medical tourism in India, specifically. As mentioned, it was actually birthed from the recommendations of a prior study, but it is unique in how and when the research occurred and the method that was used to study medical tourists who traveled to India for care. Although keeping technology and the medical industry constantly evolving, the core of this study’s findings can be beneficial to health care providers, patients, and governments. The method can be applied to research medical tourism in other countries or even to gather consumer feedback on topics beyond health care. About 25 years ago, this study would not have been possible. The advancement of internet search engines and increased socializing on the internet has greatly helped to evolve research. Now, there are actual E-Hospitals. Hong [33] wrote about the Chinese American Physician E-Hospital’s October 2015 grand opening. All physicians affiliated with this E-Hospital are bilingual Chinese American physicians who are board certified in the United States, and the website is designed specifically for the Chinese population (webpage only available in Chinese). The services provided include international transfer in the United States, saving the patients the hassle of identifying and connecting with an appropriate health service provider and also minimizing language and cultural barriers [33].

The limitations previously stated must still be considered, specifically considering the fact that there is no guaranteed way to assure all the narratives are truthful, and there is no solid estimate on how many medical tourists are actually traveling to India. However, providers hosting medical tourists in India can use the negative feedback to make corrections in the services they provide. As many patients noted disorganization was a sore point in their experience, providers can make sure to have organized lines when patients are checking in or out of the facilities. Providers who are losing patients to medical tourism can refer to this study and make service adjustments so that fewer patients are swayed to become medical tourists. For example, more providers might start practicing forms of Ayurveda, which is currently not provided in many countries outside of India. Many patients in the study specifically went to India to receive Ayurvedic care. Some narratives mentioned extending the length of trips so patients could receive appropriate follow-up care and some mentioned that follow-up care was not possible in their home countries. Providers can seek ways to enhance the follow-up care process by possibly setting up satellite offices in partnership with providers in other countries.

These results open up more options for patients. There are positives (ie, very affordable) and negatives (ie, bad traffic) about medical tourism in India. In addition, prospective patients can use altered and less-intensive versions of this study’s method to research how they can resolve their specific health issues as medical tourists in India or in other countries. In addition, patients who have previously been medical tourists and review this project can add to the details provided by authors of the narratives. More participation will help increase the accuracy of future work.

## Appendix

Multimedia Appendix 1 Steps in the Narrative Process.

Multimedia Appendix 2 Theme frequency count table.
