# Supplementary figures and images for: Internet Narratives Focused on Health Travelers’ Experiences in India: Qualitative Analysis
Source: J Med Internet Res. 2020 May 14;22(5):e15665. doi: 10.2196/15665 (PMC7256749; doi:10.2196/15665)

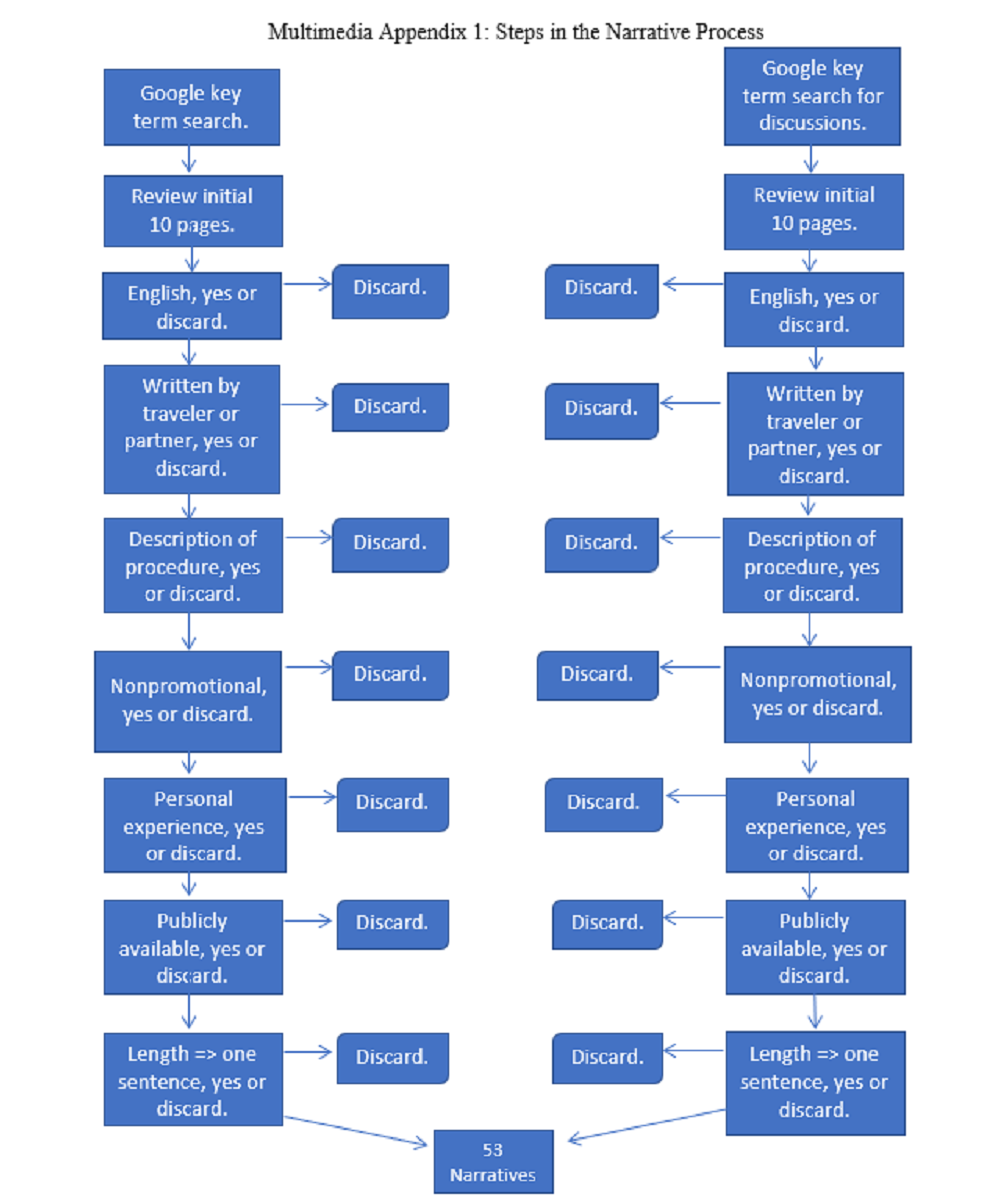

Supplement: Multimedia Appendix 1 [file jmir_v22i5e15665_app1.PNG]

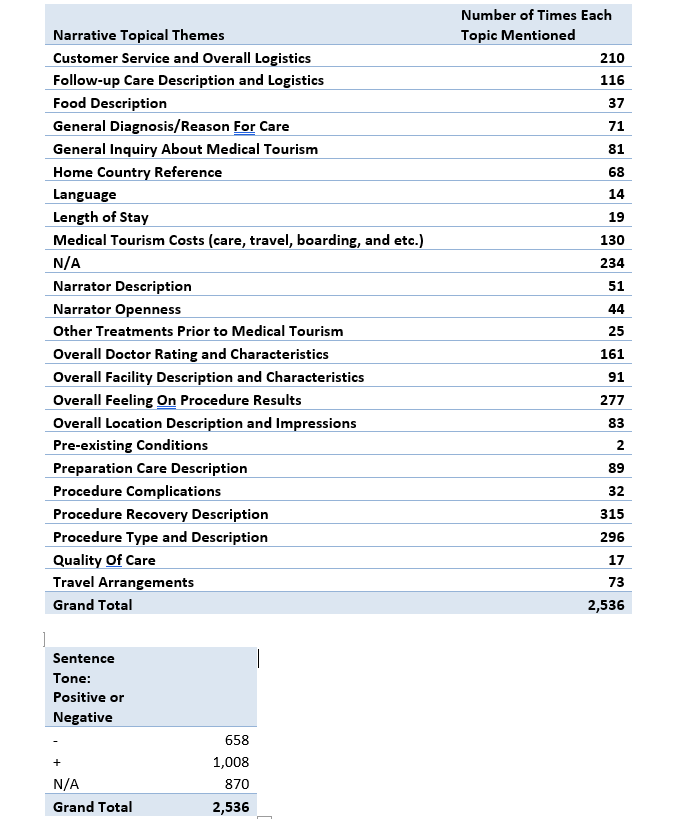

Supplement: Multimedia Appendix 2 [file jmir_v22i5e15665_app2.PNG]
